# Case Report: Mitral valve obstruction by metastatic malignant phyllodes tumor

**DOI:** 10.12688/f1000research.110022.1

**Published:** 2022-03-14

**Authors:** Chamtouri Ikram, Amdouni Nesrine, Kaddoussi Rania, Ahlem Bellalah, Kortas Chokri, Achour Asma, Joober Sameh, Maatouk Faouzi

**Affiliations:** 1 cardiology B department, Fattouma Bourguiba University Hospital, Monastir, MONASTIR, 5000, Tunisia; 2 pneumology, Fattouma Bourguiba University Hospital, Monastir, MONASTIR, 5000, Tunisia; 3 anatomic pathology, Fattouma Bourguiba University Hospital, Monastir, MONASTIR, 5000, Tunisia; 4 cardiovascular surgery, Sahloul University Hospital, MONASTIR, 5000, Tunisia; 5 radiology, Fattouma Bourguiba University Hospital, Monastir, MONASTIR, 5000, Tunisia

**Keywords:** Breast cancer, cardiac metastasis, mitral stenosis, acute heart failure

## Abstract

Cardiac metastases from phyllodes tumors (PTs) are rare. Herein, we report a case of a 37-year-old female patient with a history of borderline breast PTs, admitted to the cardiology department for acute cardiac failure revealing concomitant cardiac and pulmonary metastases of malignant PTs. Cardiac metastasis occurred through direct extension from pulmonary metastasis to the left atrium via the right inferior pulmonary vein, causing severe mitral valve obstruction. Although the metastasis was surgically removed, the patient had an uncommon complication, which led to acute heart failure and huge relapse resulting in her death.

## List of abbreviations

CT: computed tomography

LA: left atrium

LSPV: left superior pulmonary vein

MRI: magnetic resonance imaging

Pts: Phyllodes tumors

TTE: trans thoracic echocardiography

## Background

Phyllodes tumors (PTs) represent a rare category of breast neoplasm, with a prevalence accounting for <1% of all breast tumors. ^1^ PTs predominantly occur in women aged 35-50 years, ^2^ and they range from benign to malignant forms according to the histological features. ^3^ Malignant PTs account for 16% to 30% of all PTs and they have an inherent recurrence and/or metastasis potential. ^4^^,^^5^ Cardiac metastases are more frequent than primary cardiac tumors. ^6^ Herein, we report a case of concomitant cardiac and pulmonary metastases of malignant PTs, causing severe mitral valve obstruction.

## Case report

**Figure 1.**
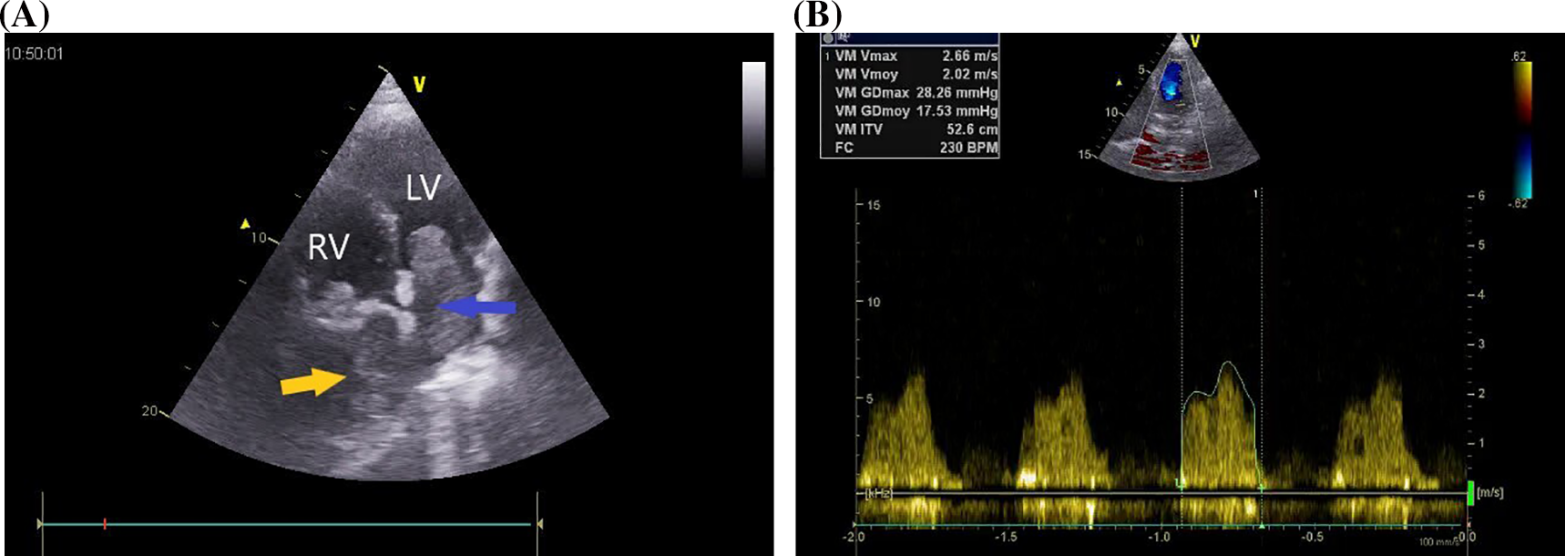
A: Transthoracic echocardiography in four-chamber view showing large mass in the left atrium (blue arrow) and a retro right atrial mass (yellow arrow). B: Transmitral valve gradient in continuous Doppler showing severe mitral stenosis. LV: left ventricle, MV: mitral valve, RV: right ventricle.

A 37-year-old Maghrebian female patient was presented to the cardiology department due to complaints of dyspnea, progressing over one month. She had a dry cough and had been resistant to symptomatic treatment. The patient was diagnosed with borderline breast PTs ten years earlier, which was treated by surgical excision. Upon examination, her dyspnea was classified as class IV on the New York Heart Association Functional Classification with orthopnea. Her transcutaneous oxygen saturation was 92%, and pulmonary auscultation revealed bibasilar crackles. Additionally, the patient’s chest x-ray showed a homogeneous opacity located in the basal part of the right lung. Transthoracic echocardiography (TTE) revealed 5 × 4 cm homogenous mass occupying nearly all the left atrium (LA), resulting in severe mitral valve obstruction (mean gradient = 17 mmHg) ( Figure 1).

**Figure 2.**
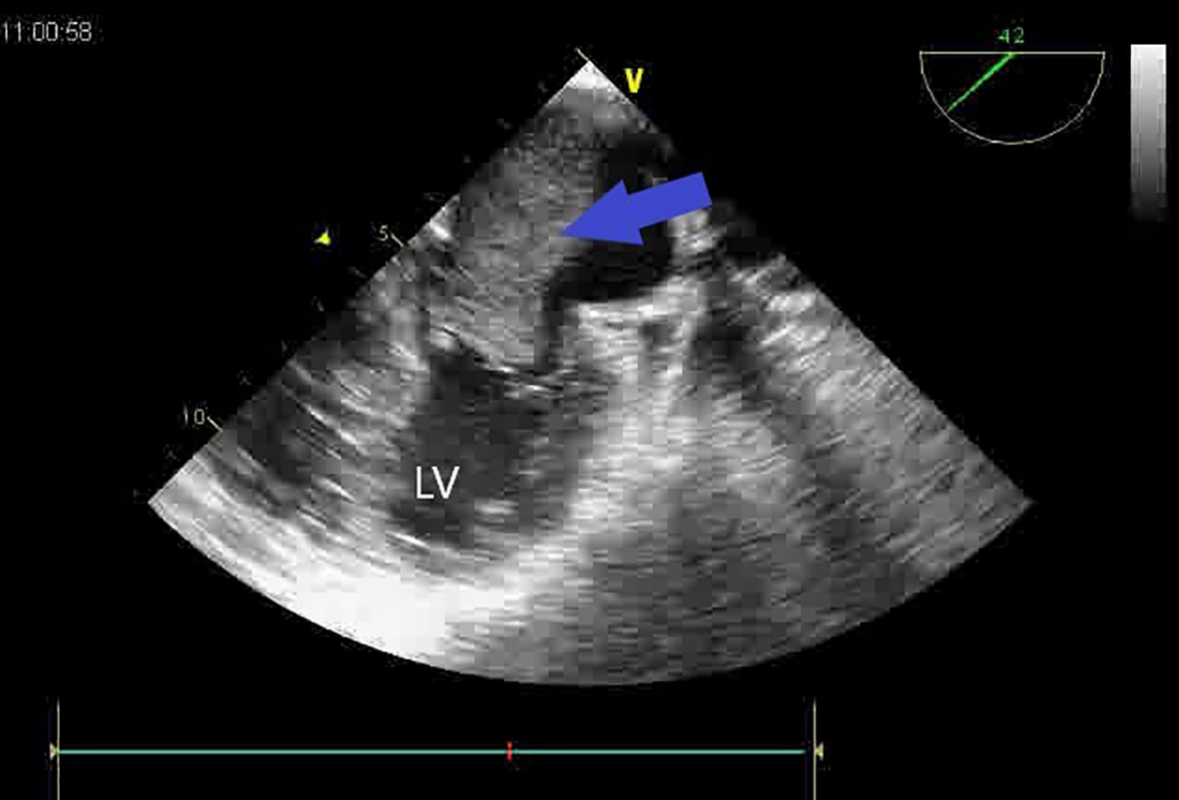
Transesopheagal echocardiography showing a large mass, occupying nearly all the left atrium (blue arrow) and mitral obstruction.

**Figure 3.**
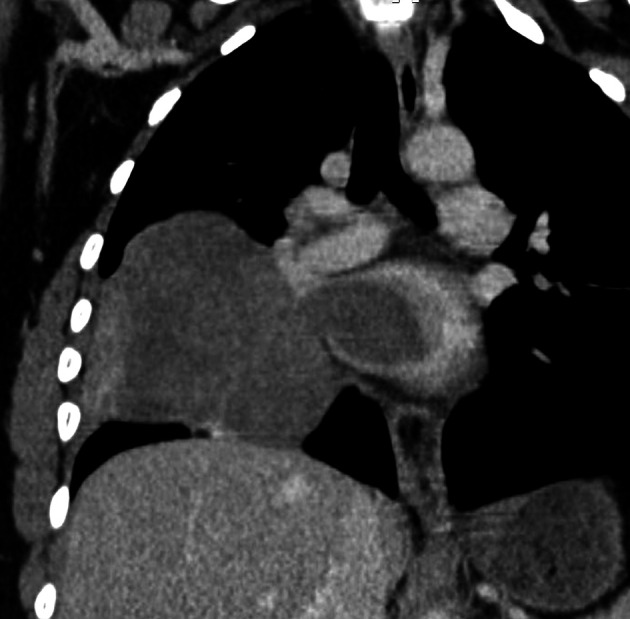
CT (coronal reconstruction): Right pulmonary mass, slightly enhanced after injection of contrast product with extension to the LA via the RIPV.

A second huge mass compressed the right atrium posterior wall. Following respiratory stabilization, transesophageal echocardiography confirmed TTE results and revealed an extended mass into LA via the right inferior pulmonary vein (RIPV) ( Figure 2). Cardiac computed tomography (CT) revealed a large (100 × 70 × 100) mediastino-pulmonary mass extending to LA via RIPV ( Figure 3).

**Figure 4.**
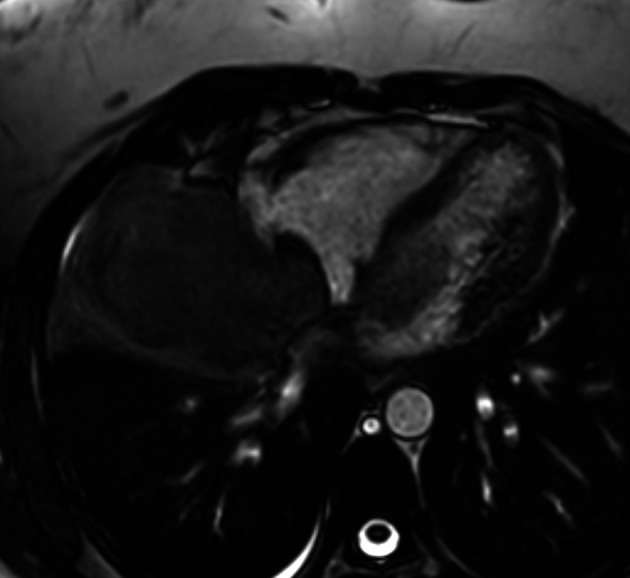
Cardiac MRI (axial cine-MRI sequence): prolapse of the mass of LA via the mitral valve.

**Figure 5.**
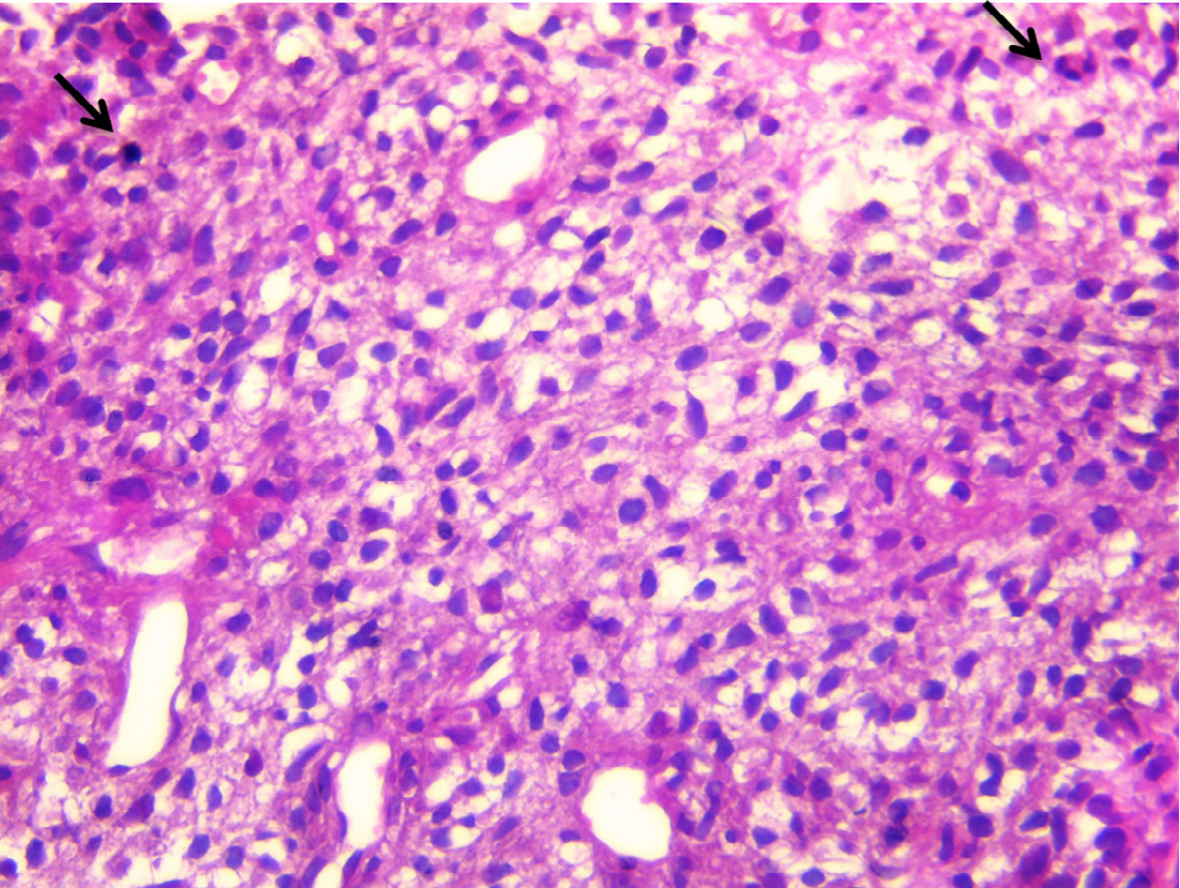
Mesenchymal pattern of a malignant phyllode tumor with a high stromal cellularity, nuclear atypia and mitosis (arrows) (HE stain × 400).

The Cardiac magnetic resonance imaging (MRI) results showed low signal on T1-weighted imaging and high signal on T2-weighted imaging of the mediastino-pulmonary mass ( Figure 4). The patient accepted to undergo an urgent mass resection surgery to avoid total mitral valve obstruction and sudden death. The histological study of the resected mass confirmed the metastatic spread of malignant PTs to LA ( Figure 5). The patient was discharged from the hospital after having an echocardiographic check-up, which demonstrated no residual tumor. However, three months after the surgery, she died from a huge relapse of mediastinal mass with tracheal invasion.

## Discussion

PTs or cystosarcoma is a rare breast neoplasm. ^1^ These types of tumors are commonly manifested in the breast tissue and are usually benign; however, they might rarely be malignant. ^2^^,^^3^ A malignant tumor has a potential to metastasize to distant organs, such as lung, bone, and liver. ^8^ Our case revealed concomitant pulmonary and cardiac metastases, which is unusual, and it is associated with poor prognosis. It has been reported that cardiac invasion could be caused by hematogenous spread, direct extension, or via the lymphatic route. ^9^ In the case of this patient, direct extension from pulmonary metastasis to RIPV is the probable route of metastasis. Reported cases of cardiac metastasis are mostly located in the right heart with the possibility of right ventricle outflow tract obstruction. ^10^ To the best of our knowledge, this is the first case of LA location, complicated by severe mitral obstruction and acute heart failure. The clinical expression of cardiac metastasis is mainly dependent on the tumor burden and location. ^6^ As in the case of our patient, cardiac metastasis can manifest with dyspnea and chest pain, or it can be asymptomatic. Previously, malignant cardiac metastasis had poor prognosis and very rare cases were identified at autopsy. ^11^ However, advances in imaging tools such as echocardiography allows for detection and confirmation of intra-cardiac mass and eventual valve or cavity obstruction. However, echocardiography is limited in the differentiation between PTs, myxoma, fibroadenoma, and thrombus. ^11^ In our case, echocardiography revealed severe mitral obstruction by an intra-LA mass. Cardiac CT and MRI provide multiple views in different axes with a precision of limits as well as intra, and extra cardiac extension, thus allowing a better distinction between the thrombus and other masses. ^12^ The results of the echocardiography, cardiac CT, and MRI for our patient confirmed the intra and extra cardiac location of the tumor and its LA access from RIPV to the mitral valve. Therapeutic approaches, including chemotherapy, radiotherapy, and hormonal therapy are still controversial. ^7^ The surgical excision of cardiac metastasis from a malignant PTs was described in few reports. ^13^ This type of intervention could be an urgent life-saving therapeutic strategy in case of right ventricle outflow obstruction or mitral obstruction, and it can also improve the patient’s quality of life in the short term, as it was in our case. ^14^^,^^15^ However, intra-operative mass manipulation could cause tumor dissemination, thus leading to a risk of further metastasis development. ^11^^,^^16^ This may explain the hudge relapse of mediastinal mass with tracheal invasion in our patient. In this case report the major limitations were the delay in diagnosing cardiac and pulmonary metastases and the lack of immunohistochemical analysis of the tumor.

## Conclusion

Cardiac metastases from PTs are rare. Tumor surgical excision might be indicated to avoid sudden death and to improve the patient’s quality of life despite the extremely unfavorable prognosis. Nevertheless, urgent surgical removal could be unavoidable in case of valve obstruction. Early diagnosis and immunohistological analysis of PTs, especially the malignant type, is imperative given that there is little effective treatment for metastatic disease.

## Data availability

All data underlying the results are available as part of the article and no additional source data are required.

## Author contributions

NA, AA and AB were actively involved in data collection and processing. IC and RK were involved in manuscript preparation. CK, SJ and FM were involved in manuscript reviewing. All authors have read and approved the manuscript.

## Consent

A written informed consent was received from the patient’s brother.
