# Supplementary material for: Case Report: Mitral valve obstruction by metastatic malignant phyllodes tumor
Source: F1000Res. 2022 Jul 25;11:309. Originally published 2022 Mar 14. [Version 2] doi: 10.12688/f1000research.110022.2 (PMC9346307; doi:10.12688/f1000research.110022.2)
Supplement: Peer review history

# Peer review: Reviewer response for version 2

**DOI:** 10.5256/f1000research.136136.r145275

**Published:** 2022-08-02

**Authors:** Chien-Liang Fang

**Affiliations:** 1 Division of Plastic and Reconstruction Surgery, Department of Surgery, Ditmanson Medical Foundation Chia-Yi Christian Hospital, Chia-Yi, Taiwan

I approve this manuscript for indexing.

Are enough details provided of any physical examination and diagnostic tests, treatment given and outcomes?

Yes

Is the case presented with sufficient detail to be useful for other practitioners?

Yes

Is sufficient discussion included of the importance of the findings and their relevance to future understanding of disease processes, diagnosis or treatment?

Partly

Is the background of the case’s history and progression described in sufficient detail?

Yes

Reviewer Expertise:

Breast and plastic surgery

I confirm that I have read this submission and believe that I have an appropriate level of expertise to confirm that it is of an acceptable scientific standard.

# Peer review: Reviewer response for version 1

**DOI:** 10.5256/f1000research.121589.r140668

**Published:** 2022-07-01

**Authors:** Chien-Liang Fang

**Affiliations:** 1 Division of Plastic and Reconstruction Surgery, Department of Surgery, Ditmanson Medical Foundation Chia-Yi Christian Hospital, Chia-Yi, Taiwan

This is a rare case of mitral valve obstruction by metastatic malignant phyllodes tumor. They provide clear and complete images for a complete preoperative assessment and they can keep patients alive for more than three months after surgery for such a severe mitral valve obstruction. They did a great job but this case report still needs further revision and correction.

 In the abstract:

1. The authors write “we report a case of a 37-year-old female patient with a history of borderline breast PTs”, please add previous breast PT surgery treatments.
2. “The patient had an uncommon complication”, please mention the complication.

In the report itself:

1. “The patient was diagnosed with borderline breast PTs ten years earlier, which was treated by surgical excision.” Which breast, tumor size, skin involvement, safe margin of surgical resection, recurrence or not?
2. What is the operation of metastatic malignant phyllodes tumor resection, including resection of mitral valve or cardiac tissue, lung resection (lobectomy) or debulking surgery? Reconstruction or not?
3. “The patient was discharged from the hospital after having an echocardiographic check-up, which demonstrated no residual tumor.” Please show the echocardiographic image.
4. “Three months after the surgery, she died from a huge relapse of mediastinal mass with tracheal invasion.”, please show the CT image.
5. In discussion, I could find more than a dozen articles on metastatic malignant phyllodes tumor of the heart, please summarize and discuss the differences between your case and theirs.
6. There are many errors in the references, please correct them.

Are enough details provided of any physical examination and diagnostic tests, treatment given and outcomes?

Yes

Is the case presented with sufficient detail to be useful for other practitioners?

Yes

Is sufficient discussion included of the importance of the findings and their relevance to future understanding of disease processes, diagnosis or treatment?

Partly

Is the background of the case’s history and progression described in sufficient detail?

Yes

Reviewer Expertise:

breast and plastic surgery

I confirm that I have read this submission and believe that I have an appropriate level of expertise to confirm that it is of an acceptable scientific standard, however I have significant reservations, as outlined above.

# Response

**Published:** 2022-07-12

**Authors:** IKRAM CHAMTOURI

**Affiliations:** Fattouma Bourguiba University Hospital, Monastir, Tunisia

Dear editorial board

 Thank you for giving me the opportunity to submit a revised draft of my manuscript. We have highlighted the changes within the manuscript. Here is a point-by-point the response to the reviewers’ comments.

 In the abstract:

 1. The authors write “we report a case of a 37-year-old female patient with a history of borderline breast PTs”, please add previous breast PT surgery treatments

 Response: changes made in the new version 

 2. The patient had an uncommon complication”, please mention the complication.

 Response: mediastinal location of metastasis

 In the report itself:

 1.“The patient was diagnosed with borderline breast PTs ten years earlier, which was treated by surgical excision.” Which breast, tumor size, skin involvement, safe margin of surgical resection, recurrence or not?

 Response: Tumor size was 8 x7 x5 cm, removed surgically with no skin involvement and safe margin of resection (added in the new version)

 2. What is the operation of metastatic malignant phyllodes tumor resection, including resection of mitral valve or cardiac tissue, lung resection (lobectomy) or debulking surgery? Reconstruction or not?

 Response: Surgery consisted on total intra cardiac metastasis resection with mitral valve conservation and right pneumonectomy without reconstruction.( added in the new version)

 3. “The patient was discharged from the hospital after having an echocardiographic check-up, which demonstrated no residual tumor.” Please show the echocardiographic image.

 Response: no echocardiographic image was available

 4. “Three months after the surgery, she died from a huge relapse of mediastinal mass with tracheal invasion.”, please show the CT image.

 Response: CT image added in the new version

 5. In discussion, I could find more than a dozen articles on metastatic malignant phyllodes tumor of the heart, please summarize and discuss the differences between your case and theirs.

 Response: changes were made in the new version

 6.There are many errors in the references, please correct them.

 Response: errors were corrected

# Peer review: Reviewer response for version 1

**DOI:** 10.5256/f1000research.121589.r139615

**Published:** 2022-06-15

**Authors:** Hassen Ibn Hadj Amor

**Affiliations:** 1 Taher Sfar university hospital, Mahdia, Tunisia; 2 Faculty of Medicine of Monastir, Monastir, Tunisia

This is an excellent case report of a young patient presenting with dyspnea class IV on the New York Heart Association functional classification, including a cascade of exploration including Transthoracic echocardiography, Transesopheagal echocardiography, CT-scan and cardiac magnetic resonance imaging as well as the histological study, concluded that the mitral valve was obstructed by a metastatic malignant phyllodes tumor. The patient required emergency valve surgery with the particularity of preserving the native valve and removing only the metastatic mass (urgent life-saving therapeutic strategy). It is a very rare cause of mitral obstruction where only the histological examination allowed to differentiate it with certainty.

 The iconography is well done. Discussion is sufficient with a good review of the literature

Are enough details provided of any physical examination and diagnostic tests, treatment given and outcomes?

Yes

Is the case presented with sufficient detail to be useful for other practitioners?

Yes

Is sufficient discussion included of the importance of the findings and their relevance to future understanding of disease processes, diagnosis or treatment?

Yes

Is the background of the case’s history and progression described in sufficient detail?

Yes

Reviewer Expertise:

Cardiology

I confirm that I have read this submission and believe that I have an appropriate level of expertise to confirm that it is of an acceptable scientific standard.
